# Prevalence of food allergy in Vietnam: comparison of web-based with traditional paper-based survey

**DOI:** 10.1186/s40413-018-0195-2

**Published:** 2018-07-23

**Authors:** Thu T. K. Le, Thuy T. B. Tran, Huong T. M. Ho, An T. L. Vu, Andreas L. Lopata

**Affiliations:** 1 Molecular Allergy Research Laboratory, College of Public Health, Medical and Veterinary Sciences, James Cook University, Townsville, Queensland Australia; 2 Centre for Biodiscovery and Molecular Development of Therapeutics, James Cook University, Townsville, Queensland Australia; 3 Australian Institute of Tropical Health and Medicine, James Cook University, Townsville, Queensland Australia; 4 Faculty of Food Technology, Nha Trang University, Khanh Hoa, Vietnam; 5 Faculty of Food Science and Technology, Ho Chi Minh City University of Food Industry, Ho Chi Minh City, Vietnam; 6 Faculty of Food Science and Technology, Nong Lam University of Ho Chi Minh City, Ho Chi Minh City, Vietnam

**Keywords:** Food allergy, Web-based survey, Paper-based survey, Population-based survey, Vietnam, Epidemiological survey, Seafood allergy, Adults, Prevalence

## Abstract

**Background:**

Web-based surveys (WBS) are increasingly applied in epidemiological studies as an appealing alternative to traditional survey methods. Rapid data collection, reduced expenditure and ease of access to large populations are some of the clear advantages of online surveys. However, WBS are still subject to limitations in terms of sample size, response rate and other additional biases compared to traditional survey methods. In the present study, we seek to validate data on food allergy (FA) in two independent sample populations collected from a WBS, and compare it to a paper-based survey (PBS).

**Methods:**

Data collected from two survey modes were compared by hypothesis testing for independent sample population. The WBS included 1185 respondents, while the PBS included 9039 respondents.

**Results:**

Overall, the data from the WBS were comparable to the PBS conducted over the same period of time in Vietnamese adults. There were no effects of different survey modes on the lifetime prevalence of doctor-diagnosed FA (5.7%; *P* = 0.7795, *β* = 0.05) and IgE-mediated FA (5.8%; *P* = 0.9590, *β* = 0.05). Both surveys showed the dominance of seafood allergy in this population (up to 2.6%), followed by beef allergy. Close correlation was seen in the patterns of FAs and different clinical symptoms. The contribution of family history of allergic diseases and place of residence to FA were confirmed in both surveys.

**Conclusions:**

The consistency of the WBS results with the PBS indicates a promising application of online surveys as an economic and validated model for future epidemiological studies, specifically in developing countries.

**Electronic supplementary material:**

The online version of this article (10.1186/s40413-018-0195-2) contains supplementary material, which is available to authorized users.

## Background

Food allergy (FA) is a growing public health concern worldwide, affecting the wellbeing and quality of life of about 4% of adults and 6% of children in the general population [1]. FA has received much attention in Western countries due to the high prevalence and severity of food-related anaphylaxis, especially in young children [2, 3]. Many of these countries have comprehensive healthcare initiatives to help manage FA, such as HealthNuts in Australia, EuroPrevall in the European community and National Health Interview Survey in the United States of America (USA). These national/multinational programmes have contributed enormously to improve the quality of life of affected people as well as raise public awareness of FA.

In other parts of the world, FA studies remain limited [1]. For example, in Asia, only a few countries have available data on FA. Although FA has been considered as a problem resulting from modern lifestyles, recent studies in Asian communities revealed high prevalence rates of FA compared to Europe and the USA, along with unique FA patterns [4]. For instance, allergies to peanut and tree nut are the most common causes of food-induced anaphylaxis and death in children from Western countries [5], whereas the frequencies of these allergies are very low in Singapore and the Philippines [6]. Furthermore, many developing countries lack FA management policies and medical readiness for appropriate interventions [7]. This raises concerns about potential impacts of FA on population health in developing countries and emerging economies.

The paucity of FA epidemiologic data in developing countries is likely due to monetary constraints. Conventional epidemiological study methods such as telephone surveys, postal surveys or interview surveys often require a good infrastructure and substantial capital funding for implementation (i.e. employment of executive staff, development of survey programs and logistics) [8]. In addition, population-based surveys are often a prolonged process, normally requiring from one to 5 years to yield the desired outcomes. The recent information technology explosion concomitant with an increase in internet penetration worldwide has resulted in the advent of web-based surveys (WBS) as a new, cost-saving survey mode [9, 10]. In the field of FA, the first WBS was conducted in Greece in 2006 with the participation of 3673 adult subjects [11]. The survey data was collected after 3 months of implementation with low investment costs. However, one of the biggest concerns with WBS is the validation of its generated data compared to traditional survey methods. Many comparative studies have been conducted assessing the benefits of the WBS in the context of cost efficiency and time management. Yet, no studies have been implemented to validate the quality of WBS data over other traditional survey types.

In the present study, we assessed the data collected from two survey modes: WBS versus paper-based survey (PBS) on FA in Vietnamese adults. The surveys were conducted at different locations throughout Vietnam to determine the contribution of environmental factors (i.e. rural vs. urban) to FA incidence in this developing country. The main outcomes of the two independent surveys were compared, including demographic features of participants, distribution of food-induced adverse reactions, prevalence of self-reported FA, doctor-diagnosed FA and IgE-mediated FA, distribution of food allergens and the association of demographic factors with FA. This study sought to evaluate the possible application of WBS for future epidemiological studies, especially in developing countries.

## Methods

### Study design

Two population-based surveys (WBS and PBS) were conducted in a similar student population aged 16–50 years to evaluate the current prevalence and pattern of FA in Vietnamese adults. Both survey modes used the same questionnaire to collect data. Study populations were randomly selected by cluster sampling method from a list of university students in two main regions: Khanh Hoa province and Ho Chi Minh City. Furthermore, these students were also divided based on specific areas they originally came from, to assess the possible impacts of environmental factors on FA incidence. Participants were invited to one survey mode only. The surveys were anonymous and voluntary for all participants. The study design and survey procedure were reviewed and approved by the Human Research Ethics Committee at James Cook University (ID: H6437).

### Paper-based FA survey

The paper-based FA survey was conducted from March to December 2016. Questionnaires were distributed to the target population and most of the answer sheets were collected on the same day. By accepting to answer the questionnaire, the participants gave their consent to the study. The response rate was calculated by dividing the number of returned questionnaires by the total distributed.

### Web-based FA survey

Students’ email addresses were randomly selected from a list of more than 35,000 participating students. These email addresses were assigned by participating universities (Gmail, supplied by Google). Official approvals for using the students’ email in this study were obtained before conducting the survey.

An invitation letter with detailed information about the study was randomly sent to 6000 email addresses from March to May 2016. By clicking an email link to the questionnaire, participants gave their consent to the study. The waiting period for collecting the first response was 2 weeks. Another reminder email was automatically sent to the participant after 2 weeks to complete the survey, with an additional waiting time of two more weeks. Participants were invited to the survey only once and asked to disregard the reminder emails if they had already completed the questionnaire.

The WBS was designed by using Google Forms. The Google account foodallergy.vn@gmail.com for this study was set up and managed by the lead investigator to collect survey responses. Each IP address could only access the questionnaire once. Survey responses were collected anonymously and saved in the designed platform. The survey responses were backed up in Microsoft Excel for further analysis. The study data were kept confidentially and only the lead investigator has access to the survey data.

### Questionnaire design

Taking into consideration that FA definition and its symptoms might not be widely understood by most Vietnamese, we designed a questionnaire to collect general information on clinical symptoms associated with food ingestion. This structured, anonymous questionnaire was modified from recent epidemiological studies conducted in Asian populations [6, 12]. The questionnaire contained two parts: part I asked the participant demographic information (i.e. age, gender and residential location) and part II contained ten questions on FA (Additional file 1: Appendix S1). The questionnaire was translated into Vietnamese; its content and translation were reviewed by the above HREC.

### Definition of FA in the surveys

According to the most recent definition established by the EAACI and the World Allergy Organization (WAO) in 2004, FA is a hypersensitivity reaction initiated by immunological mechanisms triggered by a food component and “food-induced adverse symptoms” are any abnormal clinical response that occurs following ingestion of a food or food component.

The clinical manifestation of FA upon exposure, via ingestion, inhalation or skin contact, involves a broad spectrum of symptoms including dermal, gastrointestinal and respiratory symptoms. This study was designed to collect self-reported clinical data on food-induced adverse reactions in Vietnamese adults and interpret the prevalence of FAs in this age group. The criteria to define self-reported FA, doctor-diagnosed FA and IgE-mediated FA in this survey were based on the most recent EAACI guidelines on FA and anaphylaxis [13]. Participants who answered ‘yes’ to questions 1 to 4 in part II of the questionnaire were considered to have self-reported FA. Similarly, participants who answered ‘yes’ to questions 1 to 6 were identified as the individuals with doctor-diagnosed FA. Participants who exhibited the typical symptoms for IgE-mediated FA, including hives/urticaria or angioedema or anaphylaxis reactions (i.e. drop in blood pressure, loss of consciousness, chest pain and weak pulse) after food intake [14], and answered ‘yes’ to questions 2 to 6 were considered to have IgE-mediated FA. The lifetime prevalence of self-reported FA, doctor-diagnosed FA and IgE-mediated FA was estimated.

### Statistical analysis

Survey data were imported to the IBM SPSS Statistics for Windows, version 24.0 (IBM Corp., Armonk, N.Y., USA) for statistical analysis. Continuous variables were expressed as mean ± SD. Categorical data were calculated to generate prevalence rates. The prevalence rate was calculated to provide a 95% Confidence Interval (CI) of responses to each criterion. Comparative analysis of the same variables (i.e. FA prevalence, distribution of clinical symptoms, FA triggering food groups and multivariable logistic regression analysis results) between the two survey modes was performed by either two-tailed *t-*test or *z*-test. 95% CIs were calculated to interpret the difference in proportion or odds ratios (ORs). Statistical significance was considered at a *P* value of < 0.05 for all tests.

## Results

### Comparing the demographical data between two survey modes

**Fig. 1:**
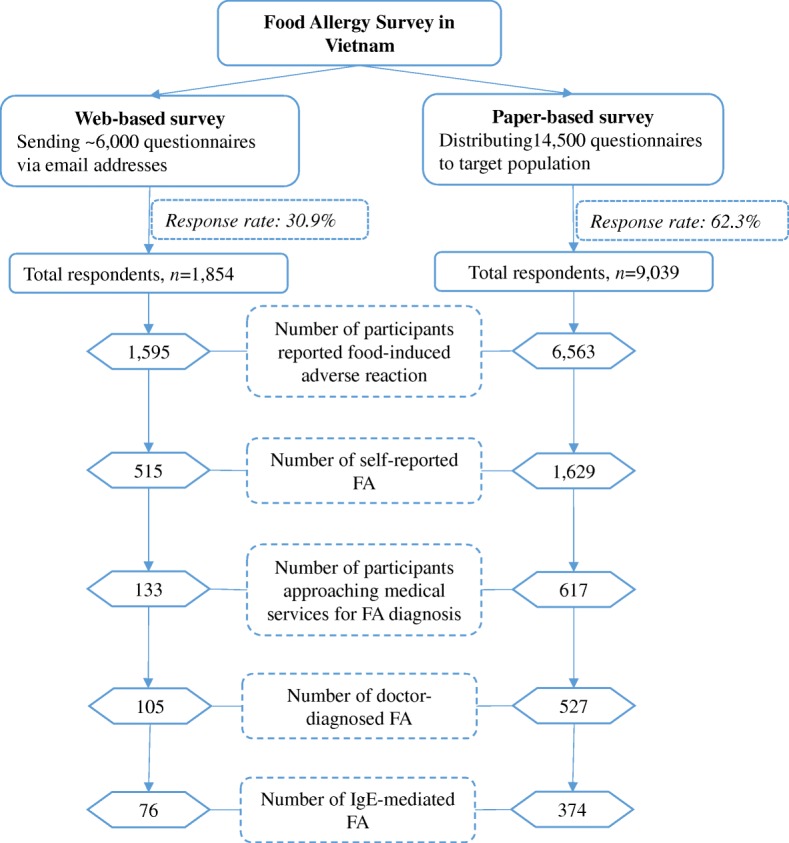
Flow diagram showing the surveys on food allergy in Vietnam. The survey was performed by two modes: web-based survey and paper-based survey

One thousand eight hundred fifty-four (1854) adult participants answered the questionnaire from the WBS compared to 9039 responses from adult participants in the PBS (Fig. 1). Overall, PBS gained a higher response rate than WBS (62.3% vs. 30.9%). The two survey modes showed the predominance of female participants: 61.7% in the WBS and 67.3% in the PBS. The average age of participants was 21.6 ± 3.4 years (WBS) and 19.8 ± 2.5 years (PBS) (Table 1).

**Table 1:** Demographic features of adult participants in the two survey modes

	Web-based survey	Paper-based survey	Difference, *P* value
Number of respondent, n	1854	9039	
Sex distribution^a^, n (%)			
Male	711 (38.3)	2955 (32.7)	< 0.001
Female	1143 (61.7)	6084 (67.3)	
Age ^b^ (years), median (minimum-maximum)	21.0 (16–47)	20.0 (18–50)	< 0.001

^a^ One Sample *t* Test was performed in SPSS Statistics 24.0 (IBM Corp., Armonk, N.Y., USA) to obtain *P* values

^b^ The Independent samples *t* Test was performed in SPSS Statistics 24.0 (IBM Corp., Armonk, N.Y., USA) to obtain *P* value

### Comparing the distribution of clinical manifestations and food triggers between the two survey modes

There were more people suffering from food-induced adverse reactions in the WBS (86.0%) than in the PBS (72.6%). The difference was seen in the number of perceived FA: 27.8% (WBS) vs. 18.0% (PBS) and the number of participants with perceived FA seeking medical advice: 25.8% (WBS) vs. 37.9% (PBS) between the two survey modes. However, the two surveys had very similar prevalence of doctor-diagnosed FA (WBS: 5.7%; PBS: 5.8%) and IgE-mediated FA (4.1% for both WBS and PBS) (Fig. 1).

**Fig. 2:**
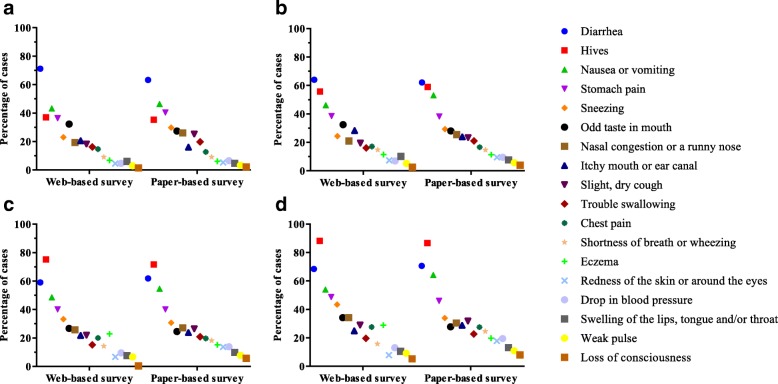
Proportion of clinical symptoms reported in two population-based survey modes. **a** Reported adverse reactions caused by food consumption in the web-based survey (*n* = 1595) and paper-based survey (*n* = 6563). **b** Reported adverse reactions in self-reported FA participant in the web-based survey (*n* = 515) and paper-based survey (*n* = 1629). **c** Reported adverse reactions in doctor-diagnosed FA participants in the web-based survey (*n* = 105) and paper-based survey (*n* = 527). **d** Reported adverse reactions in the IgE-mediated FA group in the web-based survey (*n* = 91) and paper-based survey (*n* = 433). The criteria to define IgE-mediated FA include: anaphylaxis reactions (i.e. drop in blood pressure, loss of consciousness, chest pain and weak pulse) or hives/urticaria or angioedema or anaphylaxis reactions after food intake

The proportion of clinical symptoms reported in the two surveys are presented in Fig. 2. Generally, the two study modes gained a very similar contribution of clinical symptoms in all defined groups in this study. While diarrhoea was the most common adverse symptom reported in the general study population and in the self-reported FA group, hives was the dominant symptom in doctor-diagnosed FA and IgE-mediated FA in both survey modes.

**Fig. 3:**
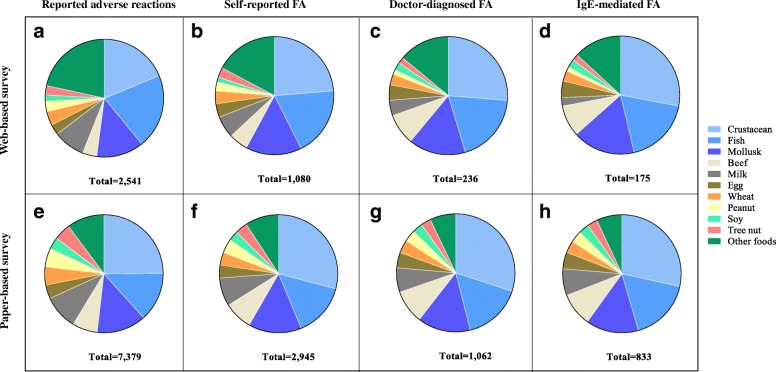
Comparison of the distribution of reported food items eliciting clinical adverse reactions in two survey modes. In the web-based survey: (**a**) Reported food-induced adverse reactions (number of participants *n* = 1595); (**b**) Self-reported FA (*n* = 515); (**c**) Doctor-diagnosed FA (*n* = 105) and (**d**) IgE-mediated FA (*n* = 91). In the paper-based survey: (**e**) Reported food-induced adverse reactions (*n* = 6563); (**f**) Self-reported FA (*n* = 1629); (**g**) Doctor-diagnosed FA (*n* = 527) and (**h**) IgE-mediated FA (*n* = 433)

In terms of triggering food items, no significant difference was seen in the contribution of food items in the surveys in regards to clinical symptoms. Seafood including fish, crustacean and shellfish stood out as the major triggering food items for food-induced adverse symptoms as well as doctor-diagnosed FA and IgE-mediated FA in both survey modes (Fig. 3 and Additional file 2: Figure S1). Minor differences were seen for other food groups, where there were more cases reported in the WBS than in the PBS.

### Comparing the prevalence of FA between the two survey modes

The prevalence of self-reported FA, doctor-diagnosed FA and IgE-mediated FA was calculated based on the defined criteria of the study (see material & method section). The prevalence rates were generated from crude data and the difference of these proportions was analyzed by two-tailed *z*-test between the two independent populations (Table 2).

**Table 2:** Prevalence of self-reported FA in Vietnam

	Self-reported FA			Doctor-diagnosed FA			IgE-mediated FA		
	Web-based survey	Paper-based survey	Difference, *P*	Web-based survey	Paper-based survey	Difference, *P*	Web-based survey	Paper-based survey	Difference, *P*
Any food	27.8 (25.7–29.8)	18.0 (17.2–18.8)	0.0000	5.7 (4.6–6.7)	5.8 (5.4–6.3)	0.7795	4.1 (3.2–5.0)	4.1 (3.7–4.6)	0.9590
Crustacean	13.8 (12.2–15.4)	9.5 (8.9–10.1)	0.0000	3.3 (2.5–4.2)	3.5 (3.2–3.9)	0.6928	2.6 (1.9–3.4	2.6 (2.3–3.0)	0.6277
Fish	11.0 (9.6–12.4)	4.8 (4.3–5.2)	0.0000	2.4 (1.7–3.1)	1.9 (1.6–2.2)	0.1233	1.7 (1.1–2.3)	1.6 (1.3–1.8)	0.3281
Mollusk	8.9 (7.6–10.2)	4.7 (4.3–5.2)	0.0000	2.0 (1.4–2.6)	1.7 (1.4–2.0)	0.3829	1.6 (1.0–2.2)	1.3 (1.1–1.6)	0.8912
Beef	3.0 (2.2–3.8)	2.5 (2.2–2.9)	0.2314	1.1 (0.6–1.6)	1.1 (0.9–1.3)	0.9829	0.8 (0.4–1.2)	0.8 (0.7–1.0)	0.0194
Milk	3.5 (2.7–4.3)	2.5 (2.2–2.9)	0.0186	0.5 (0.2–0.9)	0.8 (0.6–1.0)	0.2612	0.2 (0.0–0.4)	0.7 (0.5–0.8)	0.9465
Egg	2.2 (1.5–2.8)	1.2 (0.9–1.4)	0.0007	0.5 (0.2–0.9)	0.5 (0.4–0.6)	0.8182	0.4 (0.1–0.7)	0.4 (0.5–0.8)	0.7748
Wheat	2.1 (1.4–2.7)	1.2 (0.9–1.4)	0.0019	0.4 (0.1–0.7)	0.4 (0.3–0.6)	0.7933	0.3 (0.0–0.5)	0.3 (0.2–0.4)	0.1638
Peanut	1.4 (0.9–1.9)	1.2 (1.0–1.5)	0.5668	0.2 (0.0–0.3)	0.4 (0.3–0.5)	0.1485	0.1 (0.0–0.3)	0.3 (0.2–0.4)	0.6999
Soy	0.8 (0.4–1.2)	0.9 (0.7–1.1)	0.7485	0.3 (0.0–0.5)	0.3 (0.2–0.5)	0.6664	0.2 (0.0–0.4)	0.3 (0.2–0.4)	0.4565
Tree nut	1.6 (1.0–2.1)	1.1 (0.9–1.3)	0.0879	0.2 (0.0–0.4)	0.3 (0.2–0.4)	0.4533	0.2 (0.0–0.3)	0.3 (0.2–0.4)	0.0063
Other foods	10.0 (8.7–11.4)	3.0 (2.7–3.4)	0.0000	1.8 (1.2–2.4)	0.8 (0.6–1.0)	0.0002	1.2 (0.7–1.7)	0.6 (0.5–0.8)	0.9397

Values reported as % (95% CI).

The Two-sample *z*-test for the Difference Between Proportions was performed to obtain *P* values.

In the self-reported FA group, the two survey modes gained statistically different prevalence for most food items (*P* < 0.001), except in the cases of beef, peanut, soy and tree nut. However, in the doctor-diagnosed FA and IgE-mediated FA groups, the differences were seen in the prevalence of FA to other foods (doctor-diagnosed FA) (*P* < 0.001), as well as beef and tree nut allergy (IgE-mediated FA) (*P* < 0.01). There was no statistical evidence for the differences in FA prevalences between the two survey modes, with accepted of a type II error of 0.05. Additionally, when considering the 95% CIs of the prevalence from each variable, there was no difference in the prevalence of FAs between WBS and PBS. In summary, regardless of the survey modes and the different response rates, the WBS and PBS reported very similar prevalences of most of FAs in this study.

### The association of demographic factors with FA between the two survey modes

Multivariable logistic regression models were performed to analyze the association of demographic factors with FA (Table 3). The predictor variables were gender, family history and co-existence of other allergic diseases and outcome variable was doctor-diagnosed FA. The two-tailed *t-*test was used to compare the odds ratios of risk factors between WBS and PBS. The family history of allergy was the strongest predictor of doctor-diagnosed FA (*P* < 0.001) regardless of survey modes. There is no statistical evidence for the difference of ORs of family history as a risk factor between the two survey modes (β = 0.05). Gender and atopy conditions showed no effects on doctor-diagnosed FA in both survey modes.

**Table 3:** Multivariable logistic regression analysis of demographic factors on FA

Risk factor, (95%CI)	Web-based survey	Paper-based survey
Sex *(Female/Male)*	1.4 (0.9–2.1)	1.18 (0.97–1.44)
Family history *(Yes/No)*	4.0 (2.5–6.5)*	7.26 (5.72–9.22)*
Co-existing other allergic diseases *(Yes/No)*	1.1 (0.7–1.7)	1.09 (0.87–1.37)

‘*’ statistically significant (*P* < 0.05).

**Fig. 4:**
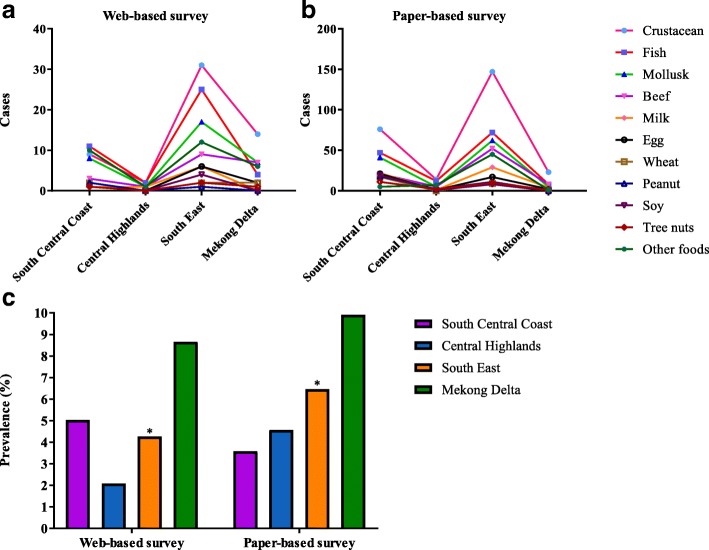
Distribution of doctor-diagnosed FAs by geographical regions of Vietnam in this study. **a** Number of doctor-diagnosed FA (*n* = 94) by triggering food items in four geographical regions in the web-based survey. **b** Number of doctor-diagnosed FA (*n* = 401) by triggering food items in four geographical regions in the paper-based survey. **c** Prevalence of FA in four geographical regions (the South Central Coast, the Central Highlands, the South East and the Mekong Delta. Asterisk ‘*’ denote significant difference in the prevalence in the South East between survey modes (*P* < 0.001). *z*-test was used to compare the two population proportions. South East is the biggest metropolitan area in Vietnam

Data on the residential locations of the survey respondents were grouped into four different geographical regions: the South Central Coast, the Central Highlands, the South East and the Mekong Delta. According to the General Statistics Office of Vietnam (GSO 2015), we defined rural and urban areas in this study taking into consideration the effects of population density, lifestyle and living environment. There are two big metropolitan areas in Vietnam: Hanoi and Ho Chi Minh City. As there were no participants from Hanoi, we grouped participants from the South East, mostly reside in Ho Chi Minh City, as people living in urban areas**.** Participants living in other parts of the country were considered to live in rural areas. A comparison was made to evaluate the impact of geographical location on FA incidence. First, we observed a higher number of doctor-diagnosed FA subjects in the South East compared to other parts of the country in both survey types (Fig. 4a-b). However, there were no statistical evidences for the difference in prevalence of doctor-diagnosed FAs among these regions between the two survey modes (β = 0.05). Only in the South East, we reported a statistically significant difference of the overall prevalence of doctor-diagnosed FA resulted from different survey modes (*P* < 0.001) (Fig. 4c).

## Discussion

This is the first study to validate data from two survey modes, WBS and traditional PBS, using the same questionnaire in an identical population. In general, the data from this WBS were comparable to the PBS conducted at the same point of time in two independent sample populations, especially with respect to the prevalence rates of FA, FA patterns and the distribution of clinical presentation.

However, we also observed substantial variations in self-reported FA prevalence between WBS (27.8%) and PBS (18.0%). This more or less reflects the current understanding of Vietnamese participants about FA definition and its clinical manifestations. In reality, the prevalence of self-reported FA might vary from 3 to 35% when comparing different epidemiological studies in the USA [15], Europe [16] and Asia [4]. However, the overall prevalence of doctor-diagnosed FA and IgE-mediated FA across the two survey modes were similar and comparable to previous studies in adults in Taiwan [17], USA and Canada [18]. Furthermore, both surveys once again confirmed the predominance of seafood allergy in Asian population [19]. Seafood accounts for more than half of the reported food-induced allergic reactions in this study, and this observation was reported previously from population-based questionnaire surveys in children in Thailand, the Philippines and Singapore [6, 20]. Additionally, very low rates of peanut, tree nut and wheat allergy were established, closely correlated to other studies performed in Asian countries [6, 21].

FA can often be confused with other non-allergic food hypersensitivities due to its wide spectrum of clinical symptoms [13]. In spite of using different survey types, we observed a very similar pattern of reported clinical symptoms among defined FA groups. Although there were more self-reported FA participants reporting gastrointestinal symptoms (diarrhoea, nausea or vomiting, stomach pain) in the WBS than in the PBS, we found no significant effect of survey modes to the outcomes of clinical manifestations. Hives was the most frequent adverse symptom for FA, followed by diarrhoea in doctor-diagnosed FA and IgE-mediated FA. The major limitation of this study was that the information of doctor-diagnosed FA was self-administered. It would be ideal to confirm the allergic responses in suspected participants by in vitro and in vivo tests. However, in Vietnam, limited services and commercial diagnostic tests are available for food allergic people, especially in rural areas. According to our knowledge, at present, there are very few allergists and allergy clinics located in the two biggest cities in Vietnam, Hanoi and Ho Chi Minh City, that could provide in vitro and in vivo FA diagnostic tests. Hence, very few people could have access to all in vitro and in vivo FA tests. Further investigations in the affected population, using a combination of above mentioned tests are initiated by our group.

The multivariable logistic regression analysis of demographic factors to FA in the two survey modes strengthens the validation of WBS with respect to PBS. Family history of allergic diseases was the strongest indicator for FA in WBS and PBS (*P* < 0.05), whilst gender and atopy condition did not have any effects. With respect to the association of geographical region to FA incidence in the two survey modes, people living in rural areas showed a lower prevalence of FA than those in urban areas. A difference in prevalence of FA between survey types was only observed in the South East. In other regions, no statistical evidence was found to support a different incidence of FA between geographical regions.

As with all epidemiologic studies, there are several pitfalls that need to be considered prior to interpreting the results of a FA survey. In the case of a WBS, limitations include recall bias, response bias, participation bias and selection bias. In this study, our target population was young Vietnamese adults attending universities. Participants from the two survey modes have very similar ages (WBS: 21.6 ± 3.4 years and PBS: 19.8 ± 2.5 years) and educational level. Thus, the recall bias would be considered equal between the two survey types.

In terms of response bias, WBS showed a lower response rate (30.9%) than PBS (62.3%). Low response rate has previously been encountered in several paper-based FA surveys. For instance, in a FA survey in the UK, the authors reported a response rate of 36% [22] whilst in a nationwide Canadian study on FA, a participation rate of 34.6% was reported [23]. In an epidemiological study, response rate is associated with study bias. Normally, investigators need to collect information from the non-response group to adjust for the final prevalence rate [18, 24]. In our PBS, we assumed that people did not answer the questionnaire merely because of their non-interest in the topic. However, this ignorance might be a result of an absence of health problems arising from food ingestion. In this case, it is essential to have proper investigation on non-response bias to generate more accurate prevalence of FA in this population. In the WBS, there are a number of potential reasons that could explain the low response rate: the survey email did not reach participants; participants did not check their email frequently; the survey email was automatically placed into the participant’s spam mailbox; the possible exhaustion of internet users to online surveys; the participants were not interested in the survey or the participants had no food-related complaints. Overall, in spite of variations in sample size and response rate, key findings on FA in Vietnamese adults are consistent between the two survey modes. This is corroborated by a recent study on FA in the US in which the authors revealed that non-respondents posed no effects on demographics and other key variables after conducting a non-response bias analysis [15].

With respect to participation bias, we observed a higher proportion of female participants compared to males in both WBS and PBS, while at the time of this study, Vietnam had an equal ratio of male and female adults aged 15 to 50 years and the ratio of male and female students is 1:1.02 [25]. The tendency that a certain gender prefers a specific mode of epidemiological survey was also seen in other population-based studies [26]. Thus, an appropriate adjustment needs to be made to generate the final prevalence rate.

A major limitation of WBS is the selection bias. WBS seem to be more feasible for young population with access to the Internet than other groups in the general population (i.e. older people, workers) [27]. In case of Vietnam, people under the age of 35 years account for 60.5% of the population [25]. Furthermore, this country has a high proportion of internet users (52.1%) compared to the average internet penetration in Asia with 45.2% [28]. Most universities provide work-domain email addresses to their students and email is the major official channel for information exchange in educational institutes in Vietnam. University students were selected as the target population for this FA survey as they represent the young population of Vietnam and there is no foreseen bias between educational levels and FA incidence. Besides, this population is better educated overall and represents frequent internet users who are more likely to check their email inbox at regular intervals and enter the survey. Selection bias can be adjusted in combination with other surveys tailoring for other age groups (i.e. children) and people with occupational allergy to obtain a more accurate prevalence of FA in a community. Apart from that, no difference in the bias between the paper-based survey and internet survey could be demonstrated [9, 29].

To increase the response rate, incentives could be considered [30]. However, the decision to use incentives and the type of incentives are dependent on the available financial capacity of the research project as well as the culture of each community where the study will be implemented. Suggestions on using incentives were mentioned elsewhere [31]. In this study, we decided not to use incentives to limit the chance that participants might enter the survey more than once and thus might be a potential thread for participation bias.

In summary, we demonstrated that WBS could provide very comparable results to the traditional PBS. The economic efficiency of WBS was confirmed in this study (Additional file 3: Table S1 and Table S2) and from previous study [26], as this survey was conducted in Vietnam, a reflection of a typical developing economy in Asia. Before this study, there was no information available about FA incidence nor national clinical guidelines on FA in Vietnam. This investigation was our first analysis of the frequency of FA in Vietnam and will be followed up by *in vivo* and *in vitro* studies in an affected population. These surveys assisted in improving the awareness about FA in Vietnam and provided important information about the current FA situation for healthcare professionals and public health policy makers. The consistence of key outcome values from WBS compared to PBS indicated the potential application of online surveys in epidemiological studies in other populations with limited capital and resources. Moreover, there are numerous available survey algorithms available, including free software that are accessible to all internet users. In our opinion, this online survey could combine with national campaigns on FA to increase awareness and understanding of FA in the general population. With the continuing rise of internet penetration in the general population, this method can be applied widely in schools and in offices. However, appropriate considerations need to be given to ensure the privacy of the respondents; the study design and the questionnaires need to be reviewed by relevant Human Research Ethics Committees.

## Conclusions

The comparable results of the WBS to PBS were validated in this study. Taking into consideration all possible biases against advantages of WBS, we suggest the application of WBS as a low-cost, time-saving, labour-efficient and convenient platform to conduct surveys on FA on a population-based scale, particularly in low income countries.

## Additional files

Additional file 1: **Appendix S1.** Survey Questionnaire for adult participants. (docx 19 kb)

Additional file 2: **Figure S1.** Distribution (%) of reported food items eliciting clinical adverse reactions in two survey modes. (docx 24 kb)

Additional file 3: **Table S1.** Comparison of budget expenditure between two survey modes (PBS and WBS) used in this study. **Table S2.** Comparison of time consumed between two survey modes (PBS and WBS) used in this study. (docx 20 kb)

## Abbreviations

FA: Food allergy
HREC: Human Research Ethics Committee
PBS: Paper-based surveys
WBS: Web-based surveys

## References

[1] Boye JI (2012). Food allergies in developing and emerging economies: need for comprehensive data on prevalence rates. Clin Transl Allergy..

[2] Gaspar Â, Santos N, Piedade S, Santa-Marta C, Pires G, Sampaio G, Arêde C, Borrego LM, Morais-Almeida M (2015). One-year survey of paediatric anaphylaxis in an allergy department. Eur Ann Allergy Clin Immunol..

[3] Grabenhenrich LB, Dolle S, Moneret-Vautrin A, Kohli A, Lange L, Spindler T, Rueff F, Nemat K, Maris I, Roumpedaki E (2016). Anaphylaxis in children and adolescents: The European Anaphylaxis Registry. J Allergy Clin Immunol..

[4] Lee AJ, Thalayasingam M, Lee BW (2013). Food allergy in Asia: how does it compare?. Asia Pac Allergy..

[5] Sasaki M, Koplin JJ, Dharmage SC, Field MJ, Sawyer SM, McWilliam V, Peters RL, Gurrin LC, Vuillermin PJ, Douglass J (2017). Prevalence of clinic-defined food allergy in early adolescence: The SchoolNuts study. J Allergy Clin Immunol..

[6] Shek LP, Cabrera-Morales EA, Soh SE, Gerez I, Ng PZ, Yi FC, Ma S, Lee BW (2010). A population-based questionnaire survey on the prevalence of peanut, tree nut, and shellfish allergy in 2 Asian populations. J Allergy Clin Immunol..

[7] Sheikh A, Sheikh Z, Roberts G, Muraro A, Dhami S, Sheikh A (2017). National clinical practice guidelines for food allergy and anaphylaxis: An international assessment. Clin Exp Allergy..

[8] Ekman A, Litton JE (2007). New times, new needs; e-epidemiology. Eur J Epidemiol..

[9] Ekman A, Dickman PW, Klint Å, Weiderpass E, Litton J-E (2006). Feasibility of using web-based questionnaires in large population-based epidemiological studies. Eu J Epidemiol..

[10] van Gelder M, Bretveld RW, Roeleveld N (2010). Web-based questionnaires: the future in epidemiology?. Am J Epidemiol..

[11] Kalogeromitros D, Makris MP, Chliva C, Sergentanis TN, Church MK, Maurer M, Psaltopoulou T (2013). An internet survey on self-reported food allergy in Greece: clinical aspects and lack of appropriate medical consultation. J Eur Acad Dermatol Venereol..

[12] Connett GJ, Gerez I, Cabrera-Morales EA, Yuenyongviwat A, Ngamphaiboon J, Chatchatee P, Sangsupawanich P, Soh SE, Yap GC, Shek LP (2012). A population-based study of fish allergy in the Philippines, Singapore and Thailand. Int Arch Allergy Immunol.

[13] Muraro A, Werfel T, Hoffmann-Sommergruber K, Roberts G, Beyer K, Bindslev-Jensen C, Cardona V, Dubois A, duToit G, Eigenmann P (2014). EAACI food allergy and anaphylaxis guidelines: diagnosis and management of food allergy. Allergy..

[14] Sicherer SH, Burks AW, Sampson HA (1998). Clinical features of acute allergic reactions to peanut and tree nuts in children. Pediatrics..

[15] Verrill L, Bruns R, Luccioli S (2015). Prevalence of self reported food allergy in US adults: 2001, 2006, and 2010. Allergy Asthma Proc.

[16] Nwaru BI, Hickstein L, Panesar SS, Muraro A, Werfel T, Cardona V, Dubois AE, Halken S, Hoffmann-Sommergruber K, Poulsen LK (2014). The epidemiology of food allergy in Europe: a systematic review and meta-analysis. Allergy..

[17] Wu TC, Tsai TC, Huang CF, Chang FY, Lin CC, Huang IF, Chu CH, Lau BH, Wu L, Peng HJ (2012). Prevalence of food allergy in Taiwan: a questionnaire-based survey. Intern Med J..

[18] Soller L, Ben-Shoshan M, Harrington DW, Fragapane J, Joseph L, St Pierre Y, Godefroy SB, La Vieille S, Elliott SJ, Clarke AE (2012). Overall prevalence of self-reported food allergy in Canada. J Allergy Clin Immunol..

[19] Lopata AL, Lehrer SB (2009). New insights into seafood allergy. Curr Opin Allergy Clin Immunol..

[20] Lao-araya M, Trakultivakorn M (2012). Prevalence of food allergy among preschool children in northern Thailand. Pediatr Int..

[21] Morita E, Chinuki Y, Takahashi H, Nabika T, Yamasaki M, Shiwaku K (2012). Prevalence of wheat allergy in Japanese adults. Allergol Int..

[22] Skypala IJ, Bull S, Deegan K, Gruffydd-Jones K, Holmes S, Small I, Emery PW, Durham SR (2013). The prevalence of PFS and prevalence and characteristics of reported food allergy; a survey of UK adults aged 18–75 incorporating a validated PFS diagnostic questionnaire. Clin Exp Allergy..

[23] Ben-Shoshan M, Harrington DW, Soller L, Fragapane J, Joseph L, St Pierre Y, Godefroy SB, Elliott SJ, Clarke AE (2010). A population-based study on peanut, tree nut, fish, shellfish, and sesame allergy prevalence in Canada. J Allergy Clin Immunol..

[24] Kessler RC, Heeringa SG, Colpe LJ, Fullerton CS, Gebler N, Hwang I, Naifeh JA, Nock MK, Sampson NA, Schoenbaum M (2013). Response bias, weighting adjustments, and design effects in the Army Study to Assess Risk and Resilience in Servicemembers (Army STARRS). Int J Methods Psychiatr Res..

[25] Vietnam GSOo. Statistical Yearbook of Vietnam 2015 - Population and Employment. In*.*, 2016 edn. Vietnam: Statistical Publishing House; 2016.

[26] Smith B, Smith TC, Gray GC (2007). When epidemiology meets the Internet: Web-based surveys in the Millennium Cohort Study. Am J Epidemiol..

[27] Bech M, Kristensen MB (2009). Differential response rates in postal and Web-based surveys in older respondents. Survey Research Methods..

[28] Internet World Stats. Asia. 2017. http://www.internetworldstats.com/asia.htm#vn. Accessed 14 Aug 2017.

[29] Mayr A, Gefeller O, Prokosch H-U, Pirkl A, Fröhlich A, de Zwaan M (2012). Web-based data collection yielded an additional response bias—but had no direct effect on outcome scales. J Clin Epidemiol..

[30] Sasaki M, Yoshida K, Adachi Y, Furukawa M, Itazawa T, Odajima H, Saito H, Hide M, Akasawa A (2016). Environmental factors associated with childhood eczema: Findings from a national web-based survey. Allergol Int..

[31] Singer E, Couper MP (2008). Do incentives exert undue influence on survey participation? Experimental evidence. J Empir Res Hum Res Ethics.

