# Supplementary material for: Prevalence of food allergy in Vietnam: comparison of web-based with traditional paper-based survey
Source: World Allergy Organ J. 2018 Jul 23;11(1):16. doi: 10.1186/s40413-018-0195-2 (PMC6055338; doi:10.1186/s40413-018-0195-2)
Supplement: Supplementary file 1 — Appendix S1. Survey Questionnaire for adult participants. (docx 19 kb) [file 40413_2018_195_MOESM1_ESM.docx]

**Supplementary information**

**Appendix S1. Survey Questionnaire for adult participants**

1. **General information of respondent:**

Gender: □ Male □ Female

Age:

Living location:

1. **Questions:**

1. Have you ever had any symptoms as below when consuming foods (*tick which apply*)

□ Hives (reddish, swollen, itchy areas on the skin)

□ Eczema (a persistent dry, itchy rash)

□ Redness of the skin or around the eyes

□ Itchy mouth or ear canal

□ Nausea or vomiting

□ Diarrhea

□ Stomach pain

□ Nasal congestion or a runny nose

□ Sneezing

□ Slight, dry cough

□ Odd taste in mouth

□ Obstructive swelling of the lips, tongue, and/or throat

□ Shortness of breath or wheezing

□ Trouble swallowing

□ Drop in blood pressure

□ Loss of consciousness

□ Chest pain

□ A weak pulse

□ No symptoms as above

2. Do any symptoms as above repeat when you eat a specific food?

□ Yes □ No

3. According to your observations, the cause of any allergy-like symptoms manifests, as listed in question 1, after eating which food group below?

□ Crustacean (shrimp, crab, ………………………………………………)

□ Fish (Please specify: ……………….…………………………………)

□ Molluscs (squid, octopus, clam, snail…………………………………….)

□ Egg

□ Wheat, wheat-based products

□ Peanut

□ Soy bean

□ Tree nut: cashew, walnut, almond

□ Milk and dairy products

□ Beef meat

□ Other food commodities (Please specify:…………………………………)

□ No allergy to any foods

4. Do you think that you have suffered **food allergy**?

□ Yes 🡪 please go to question 5

□ No 🡪 please go to question 7

5. Have you ever visited specialized doctor for food allergy?

□ Yes □ No

6. Have you been diagnosed to have **food allergy**?

□ Yes □ No

7. Are you or is there any other member in your family have **other types of allergy** (pollen allergy, antibiotics allergy…)?

□ Yes □ No

8. Are you or is there any other member in your family have **food allergy**?

□ Yes □ No

9. If yes, please specify the food that you or other members in your family are allergic to:

………………………………………………………………………………………………………………………………………………………………………………………………………………………………………………………………………………

10. If you have been suffering any of the above symptoms of food allergies, do you wish to follow up the second phase of the project to investigate the food allergy causative factors?

□ Yes □ No
