# Supplementary material for: Prevalence of food allergy in Vietnam: comparison of web-based with traditional paper-based survey
Source: World Allergy Organ J. 2018 Jul 23;11(1):16. doi: 10.1186/s40413-018-0195-2 (PMC6055338; doi:10.1186/s40413-018-0195-2)
Supplement: Supplementary file 2 — Figure S1. Distribution (%) of reported food items eliciting clinical adverse reactions in two survey modes. (docx 24 kb) [file 40413_2018_195_MOESM2_ESM.docx]

**Figure S1**: Distribution (%) of reported food items eliciting clinical adverse reactions in two survey modes

| **Food item** | **Reported adverse reactions (%)** | | **Self-reported FA (%)** | | **Doctor-diagnosed FA (%)** | | **IgE-mediated FA(%)** | |
| --- | --- | --- | --- | --- | --- | --- | --- | --- |
|  | **WBS** | **PBS** | **WBS** | **PBS** | **WBS** | **PBS** | **WBS** | **PBS** |
| Crustacean | 18.7 | 24.9 | 23.7 | 29.1 | 26.3 | 30.0 | 28.0 | 28.5 |
| Fish | 20.4 | 13.5 | 18.9 | 14.6 | 19.1 | 16.0 | 18.3 | 17.0 |
| Mollusk | 12.9 | 13.5 | 15.3 | 14.5 | 15.7 | 14.5 | 17.1 | 14.4 |
| Beef | 3.9 | 6.8 | 5.2 | 7.8 | 8.5 | 9.1 | 8.6 | 9.1 |
| Milk | 8.7 | 9.5 | 6.0 | 7.8 | 4.2 | 6.7 | 2.3 | 7.3 |
| Egg | 2.9 | 3.8 | 3.7 | 3.6 | 4.2 | 4.2 | 4.6 | 4.6 |
| Wheat | 3.9 | 5.0 | 3.5 | 3.5 | 3.0 | 3.6 | 2.9 | 3.4 |
| Peanut | 2.8 | 5.0 | 2.4 | 3.8 | 1.3 | 3.2 | 1.1 | 3.1 |
| Soy | 1.8 | 3.3 | 1.4 | 2.7 | 2.1 | 2.8 | 2.3 | 2.9 |
| Tree nut | 2.5 | 4.6 | 2.7 | 3.4 | 1.7 | 2.7 | 1.7 | 2.8 |
| Other foods | 21.6 | 10.2 | 17.2 | 9.2 | 14.0 | 7.1 | 13.1 | 7.0 |

WBS: web-based survey

PBS: paper-based survey
