# Supplementary material for: Prevalence of food allergy in Vietnam: comparison of web-based with traditional paper-based survey
Source: World Allergy Organ J. 2018 Jul 23;11(1):16. doi: 10.1186/s40413-018-0195-2 (PMC6055338; doi:10.1186/s40413-018-0195-2)
Supplement: Supplementary file 3 — Table S1. Comparison of budget expenditure between two survey modes (PBS and WBS) used in this study. Table S2. Comparison of time consumed between two survey modes (PBS and WBS) used in this study. (docx 20 kb) [file 40413_2018_195_MOESM3_ESM.docx]

**Table S1.** Comparison of budget expenditure between two survey modes (PBS and WBS) used in this study

| **Cost breakout** | **Paper-based survey** | **Web-based survey** |
| --- | --- | --- |
| Printing out survey questionnaires | Yes | None |
| Transportation cost of survey materials to the survey sites and returning the questionnaires | Yes | None |
| Cost involved organising the survey campaign, distributing the survey materials and collecting the survey answer sheets (labour cost, stationery cost, meals …) | Yes | None |
| Data input cost (labour cost to transfer the survey data into Microsoft Excel for data analysis) | Yes | None |
| Data input cross-check (labour cost to cross-check the input survey data) | Yes | None |
| Storage cost of survey answer sheets | Yes | None |

**Table S2**. Comparison of time consumed between two survey modes (PBS and WBS) used in this study

| **Time consumption breakout** | **Paper-based survey** | **Web-based survey** |
| --- | --- | --- |
| Preparing survey material (printing, logistics) | 60 hours | 5 hours |
| Distributing the survey questionnaire to the survey sites and collecting the survey answer sheets | 120 hours | None |
| Inputting data survey into Microsoft Excel and SPSS for data analysis | An average of 5 minutes per answer sheet * ~9,040 documents = 754 hours | At the end of the survey, the data in the Google Form spreadsheet was copied to a Microsoft Excel spreadsheet for data analysis. This activities took about 1 hour. |
| Cross-checking data and correcting mistakes | About 250 hours | We did cross-check data to make sure that all the data were accurately copied and analysed. This activity took about 6 hours. |
| Data analysis | 60 hours | 50 hours |

Note: the time consumption was calculated by the total time consumed from all people engaged in these activities.
